# Rapid Serological Assays and SARS-CoV-2 Real-Time Polymerase Chain Reaction Assays for the Detection of SARS-CoV-2: Comparative Study

**DOI:** 10.2196/19152

**Published:** 2020-10-30

**Authors:** Angelo Virgilio Paradiso, Simona De Summa, Daniela Loconsole, Vito Procacci, Anna Sallustio, Francesca Centrone, Nicola Silvestris, Vito Cafagna, Giuseppe De Palma, Antonio Tufaro, Vito Michele Garrisi, Maria Chironna

**Affiliations:** 1 Science Direction IRCCS Istituto Tumori Giovanni Paolo II Bari Italy; 2 Molecular Diagnostics and Pharmacogenetics Unit IRCCS Istituto Tumori Giovanni Paolo II Bari Italy; 3 Department of Biomedical Sciences and Human Oncology-Hygiene Section University of Bari Bari Italy; 4 Emergency Department Policlinico Hospital Bari Italy; 5 Regional Epidemiological Observatory Apulia Region Bari Italy; 6 Unit of Internal Medicine Guido Baccelli Department of Biomedical Sciences and Human Oncology University of Bari Medical School Bari Italy; 7 Medical Oncology Unit IRCCS Istituto Tumori Giovanni Paolo II Bari Italy; 8 Clinical Pathology Laboratory IRCCS Istituto Tumori Giovanni Paolo II Bari Italy; 9 Experimental Oncology and BioBank Management Unit Institutional BioBank IRCCS Istituto Tumori Giovanni Paolo II Bari Italy; 10 Hygeine Unit Policlinico Hospital Bari Italy

**Keywords:** SARS-CoV-2, COVID-19, serological test, RT-PCR

## Abstract

**Background:**

Real-time polymerase chain reaction (RT-PCR) testing for the identification of viral nucleic acid is the current standard for the diagnosis of SARS-CoV-2 infection, but technical issues limit its utilization for large-scale screening. Serological immunoglobulin M (IgM)/IgG testing has been proposed as a useful tool for detecting SARS-CoV-2 exposure.

**Objective:**

The objective of our study was to compare the results of the rapid serological VivaDiag test for SARS-CoV-2–related IgM/IgG detection with those of the standard RT-PCR laboratory test for identifying SARS-CoV-2 nucleic acid.

**Methods:**

We simultaneously performed both serological and molecular tests with a consecutive series of 191 symptomatic patients. The results provided by a new rapid serological colorimetric test for analyzing IgM/IgG expression were compared with those of RT-PCR testing for SARS-CoV-2 detection.

**Results:**

Of the 191 subjects, 70 (36.6%) tested positive for SARS-CoV-2 based on RT-PCR results, while 34 (17.3%) tested positive based on serological IgM/IgG expression. Additionally, 13 (6.8%) subjects tested positive based on serological test results, but also tested negative based on RT-PCR results. The rapid serological test had a sensitivity of 30% and a specificity of 89% compared to the standard RT-PCR assay. Interestingly, the performance of both assays improved 8 days after symptom appearance. After 10 days had passed since symptom appearance, the predictive value of the rapid serological test was higher than that of the standard molecular assay (proportion of positive results: 40% vs 20%). Multivariate analysis showed that age >58 years *(P*<.01) and period of >15 days after symptom onset (*P*<.02) were significant and independent factors associated with serological test positivity.

**Conclusions:**

The rapid serological test analyzed in this study seems limited in terms of usefulness when diagnosing SARS-CoV-2 infection. However, it may be useful for providing relevant information on people’s immunoreaction to COVID-19 exposure.

## Introduction

Recently, a novel coronavirus, which was first reported in China, capable of interperson transmission has been causing lethal pneumonia in humans [1]. Subsequent molecular studies confirmed that the origin of this transmissible pneumonia was the novel SARS-CoV-2 virus, which causes the new COVID-19 disease [2].

As the COVID-19 disease rapidly spread to other Asian and European countries, the Italian Government had to take drastic measures to contain the outbreak, including establishing strict criteria to define patients from whom oropharyngeal swabs should be collected for the molecular polymerase chain reaction (PCR) diagnosis of COVID-19 and quarantining individuals who may have been in contact with SARS-CoV-2–infected people [3]. These measures were active for weeks, during which the number of new SARS-CoV-2 infection cases in Italy continued to increase, with more than 4000 new cases being reported daily [4]. Several attempts have been made to interpret the epidemiological trend of COVID-19 in Italy, and experts have focused on the limitations of early SARS-CoV-2 infection diagnosis [5] and the detection of SARS-CoV-2 infection in asymptomatic people [6].

The real-time PCR (RT-PCR) test for identifying viral nucleic acid is the current standard for the diagnosis of COVID-19. However, this assay has some practical limitations [3], such as the unpleasantness of obtaining biological material from the nasopharynx, the relatively long time required to generate results, and the need for certified laboratories and specific expertise. These limitations make RT-PCR unsuitable for quick and simple patient screening. Therefore, the search for a precise, rapid, simple, and large-scale screening test for quickly identifying SARS-CoV-2–infected patients has become urgent to prevent virus transmission and ensure timely treatment of patients.

The Saw Swee Hock School of Public Health at the National University of Singapore recently reviewed the diagnostic tests for COVID-19 infection currently undergoing clinical validation, including dozens of assays based on RT-PCR, next-generation sequencing, and microfluidics [7]. Additionally, 12 immunoassays based on evidence that COVID-19 is related to immunoglobulin G (IgG) and immunoglobulin M (IgM) expression were also listed. It has been argued that, based on previous experiences with viral SARS infection epidemics, specific IgM antibodies against SARS-CoV-2 could be detected in blood by performing immunoassays 3-6 days after symptom onset, while IgG detection could occur some days later [4]. It has also been speculated that, since SARS-CoV-2 belongs to the same large family of viruses that caused the Middle East Respiratory Syndrome and SARS epidemics, SARS-CoV-2 antibody seroconversion should be similar to that of other coronaviruses [5].

A report from the National University of Singapore has described the VivaDiag SARS-CoV-2 IgM/IgG Rapid Test kit as an immunoassay with available information regarding sensitivity and specificity [8] and a potential candidate for reliable and rapid (15 minutes) testing, according to the preliminary data available [6]. The test is based on the utilization of antihuman IgG and IgM against the recombinant antigen that represents the receptor-binding domain of the COVID-19 spike protein.

The aim of our study was to compare the results provided by the rapid serological VivaDiag test with those of standard RT-PCR testing for SARS-CoV-2 detection in swab specimens. The two tests were simultaneously performed on subjects with COVID-19 symptoms. We setup a prospective, mono-institutional, ad hoc, blinded, and independent study that enrolled a series of 191 subjects who were admitted to the Emergency Department of the Policlinico University Hospital in Bari, Italy.

## Methods

### Recruitment

Between March 23, 2020 and March 29, 2020, we enrolled a consecutive cohort of 191 patients who were admitted to the Emergency Department of the Policlinico University Hospital in Bari, Italy for COVID-19–related symptoms or because they were quarantined for previous exposure to COVID-19–positive individuals. Oropharyngeal swabs for standard SARS-CoV-2 RT-PCR analysis and venous blood samples for VivaDiag tests were simultaneously collected from each subject, and the tests were immediately performed in reference laboratories. Registries containing patients’ main clinical data, including date of symptom onset (self-reported), were created. Informed written consent was obtained from all patients. Oropharyngeal swab samples were immediately analyzed for SARS-CoV-2 by RT-PCR at the Laboratory of Molecular Epidemiology and Public Health of the Hygiene Unit of the Policlinico University Hospital (Bari, Italy), the regional reference laboratory for SARS-CoV-2 identification. The venous blood samples were analyzed at the Clinical Pathology Laboratory (Certified ISO-9001/2015; Head E. Savino) and the Institutional BioBank (Certified ISO-9001/2015; Head A. Paradiso) of the IRCCS Istituto Tumori Giovanni Paolo II (Bari, Italy). This study was approved by the Ethical Committee of the IRCCS Istituto Tumori Giovanni Paolo II, Bari (Protocol number CE 870/2020).

### Molecular Detection of SARS-CoV-2

Nasopharyngeal/oropharyngeal swabs were subjected to nucleic acid extraction with the MagNA Pure System (Roche Diagnostics), in accordance with the manufacturer’s instructions. The presence of the *E* gene, *RdRP* gene, and *N* gene of the SARS-CoV-2 virus were identified by a commercial RT-PCR assay (Allplex 2019-nCoV Assay; Seegene). Samples were considered positive at molecular screening if all three genes were detected. The Centers for Disease Control and Prevention RT-PCR protocol was used to confirm the presence of SARS-CoV-2 [9]. To date [10,11], this methodology is considered the gold standard for the detection of SARS-CoV-2 infection.

### SARS-CoV-2 IgM/IgG Rapid Test

The SARS-CoV-2 IgM/IgG combined antibody rapid test kit, VivaDiag, (VivaChek Biotech) is a lateral flow qualitative immunoassay used for the rapid determination of the presence or absence of both anti-SARS-CoV-2-IgM and anti-SARS-CoV-2-IgG in human specimens (whole blood, serum, and plasma). A surface antigen from SARS-CoV-2, which can specifically bind to SARS-CoV-2 antibodies (including both IgM and IgG), is conjugated to colloidal gold nanoparticles and sprayed onto conjugation pads. The rapid SARS-CoV-2 IgG/IgM combined antibody test strip has two mouse antihuman monoclonal antibodies (anti-IgG and anti-IgM) on two separate test lines.

When testing, 10-15 µL of a specimen was inserted into the sample port, and then the sample dilution buffer was added. As the specimen flowed through the device, anti-SARS-CoV-2 IgG and IgM antibodies, if present in the specimen, were bound by the SARS-CoV-2 antigens (ie, the gold colorimetric reagent fixed on the conjugate pad). As the conjugated sample continued to travel up the strip, the anti-SARS-CoV-2 IgM antibodies were bound on the M (IgM) line, and the anti-COVID-19 IgG antibodies were bound to the G (IgG) line. If the specimen did not contain SARS-CoV-2 antibodies, no labeled complexes were bound. The presence of SARS-CoV-2 IgG and IgM antibodies was indicated by a red/purple line on a specific region of the device. Each test was evaluated by two readers, and a picture was taken of the result. In case of disagreement, the picture was evaluated by a third party.

### Statistical Analysis

The performance of the VivaDiag tests was compared to that of the RT-PCR tests using the caret R package, which computed all the parameters needed (sensitivity, specificity, accuracy, and Cohen κ). The performance evaluation was carried out using RT-PCR as the gold standard. Both tests were performed on the same subjects. Univariate and multivariate logistic regression were performed. Age was dichotomized by using the median age as a cutoff, and the number of days after the onset of symptoms was used as a categorical variable (0-5 days, 6-8 days, 9-10 days, 11-15 days, >15 days). All analyses were carried out in R version 3.6 (The R Foundation), and results were considered to be significant when the *P* value was <.05.

## Results

All 191 subjects enrolled in the study underwent a SARS-CoV-2 RT-PCR test and IgM/IgG rapid test. The cohort had a median age of 58.5 years, and 60.6% (116/191) were male. Subjects were admitted to the emergency room at different times after the onset of symptoms. A description of symptoms was available for 160 (83.8%) of the 191 patients. Of these 160 subjects, 14 (8.7%) were in quarantine and asymptomatic at the time they arrived to the emergency room.

**Figure 1:**
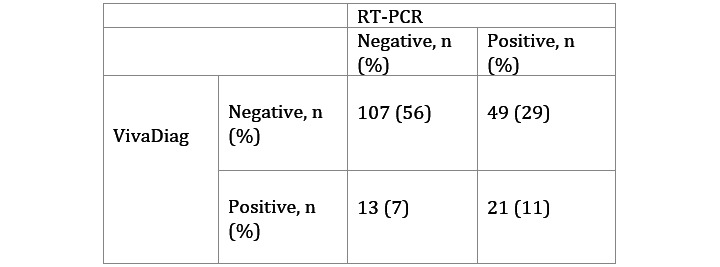
Comparison of RT-PCR and VivaDiag results from a series of 191 subjects (*P*=.001). Compared to RT-PCR, VivaDiag had a sensitivity of 30%, specificity of 89%, accuracy of 67% (95% CI 60-74), and Cohen κ value of 0.21. RT-PCR: real-time polymerase chain reaction.

**Figure 2:**
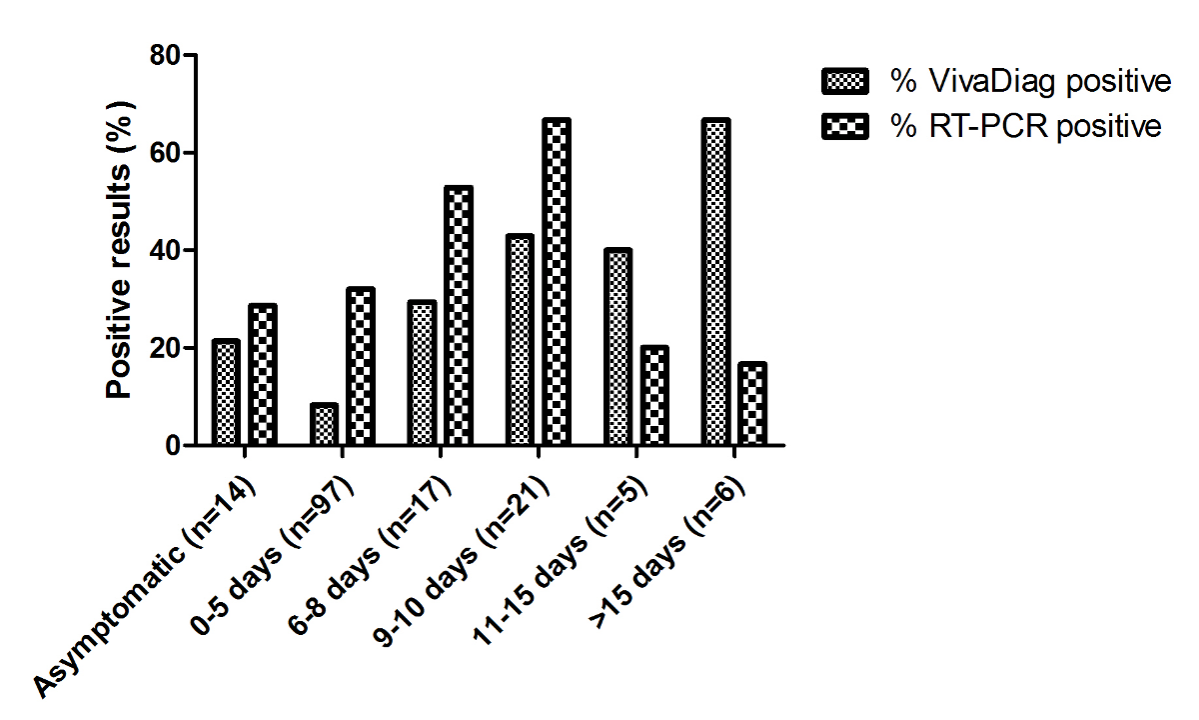
Bar plot depicting the distribution of the proportion of positive results from the VivaDiag serological test and those from RT-PCR testing for SARS-CoV-2 detection on oropharyngeal swab specimens according to time after symptom onset to test performance. RT-PCR: real-time polymerase chain reaction.

Of the 191 patients, 70 (36.6%) tested positive for SARS-CoV-2 based on RT-PCR results, while 34 (17.8%) tested positive based on serum IgM/IgG rapid test results. Compared to the RT-PCR test, the serological test had an accuracy of 67% (95% CI 60-74), a sensitivity of 30%, and a specificity of 89%. The Cohen κ value was 0.21, meaning that the strength of agreement was, according to Altman [12], considered fair. Notably, 13 patients (6.8%) tested positive based on serological test results, but also tested negative based on RT-PCR results (Figure 1). Of these 13 subjects, 7 (54%) obtained positive IgG/IgM test results at different times after symptom onset (range 10-27 days), while 6 (46%) only obtained positive IgM results at various times after symptom appearance (range 4-25 days). The distribution of the percentage of positive results detected by both tests broken down by days from symptom onset is shown in Figure 2.

A clear increase in the number of positive serological tests was observed as more days elapsed from symptom appearance, reaching 66.7% at 15 days after symptom onset. Conversely, the highest likelihood for a positive RT-PCR test result was seen from 9 to 10 days after symptom onset, and it decreased rapidly afterwards over time. Of the 14 asymptomatic individuals, 4 (29%) had positive RT-PCR test results, while only 1 (7%) had a positive serological test result.

**Figure 3:**
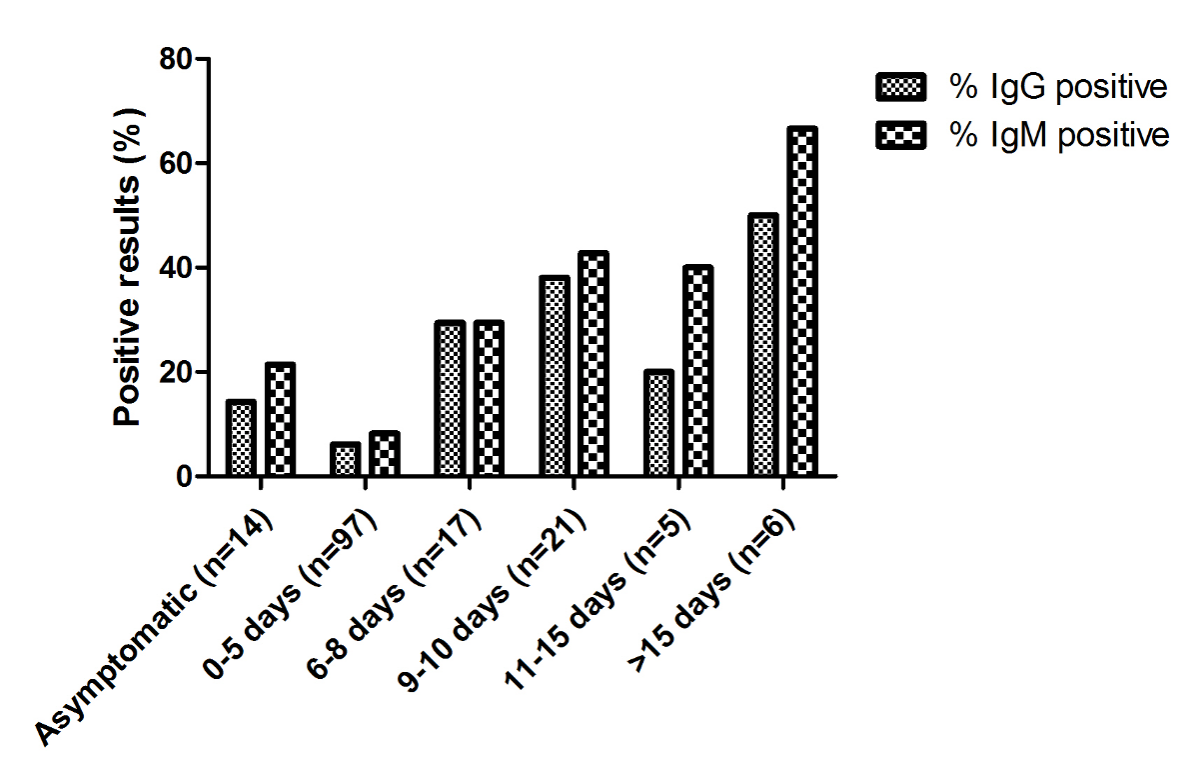
Bar plot depicting the distribution of the proportion of positive IgG and IgM VivaDiag test results according to time after symptom onset to test performance. IgG: immunoglobulin G; IgM; immunoglobulin M.

Further analysis regarding the behavior of IgM and IgG according to the time after symptom onset is described in Figure 3. Only minimal differences in the behavior of the two immunoglobulins with respect to the time of symptom appearance became evident. However, all 13 patients with positive VivaDiag tests and negative RT-PCR results had positive IgM results, while only 7 (54%) of them also had positive IgG results.

Univariate and multivariate logistic regressions were performed to identify independent predictive variables for positive VivaDiag and RT-PCR test results (Tables 1-2). Both univariate and multivariate analyses showed that age >58.5 years and period of >15 days from symptom onset were significantly associated with VivaDiag positivity, while 9-10 days after symptom onset was independently associated with a positive RT-PCR test result.

**Table 1:** Univariate and multivariate logistic regression results using VivaDiag positivity as a dependent variable.

Variable				OR (95% CI)		*P* value
**Univariate logistic regression analysis**						
	**Days after symptom onset**					
		Asymptomatic	Ref		Ref	
		0-5	0.32 (0.08-1.66)		.13	
		6-8	1.52 (0.3-8.9)		.61	
		9-10	2.74 (0.63-14.88)		.19	
		11-15	2.44 (0.23-23.39)		.42	
		>15	7.33 (0.96-77.28)		.06	
	**Age (years)**					
		≤58.5	Ref		Ref	
		>58.5	2.99 (1.31-7.31)		.01	
	**Sex**					
		Female	Ref		Ref	
		Male	1.22 (0.55-2.85)		.62	
**Multivariate logistic regression analysis**						
	**Days after symptom onset**					
		>15	12.3 (1.44-148.14)		.02	
	**Age (years)**					
		≤58.5	Ref		Ref	
		>58.5	3.59 (1.39-10.48)		.01	

**Table 2:** Univariate and multivariate logistic regression results using real-time polymerase chain reaction SARS-CoV-2 positivity as a dependent variable.

Variable				OR (95% CI)		*P* value
**Univariate logistic regression analysis**						
	**Days after symptom onset**					
		Asymptomatic	Ref		Ref	
		0-5	1.17 (0.36-4.54)		.79	
		6-8	2.81(0.65-13.75)		.17	
		9-10	4.99 (1.21-24.11)		.03	
		11-15	0.62 (0.02-6.17)		.71	
		>15	0.53 (0.02-4.64)		.57	
	**Age (years)**					
		≤58.5	Ref		Ref	
		>58.5	0.89 (0.47-1.7)		.74	
	**Sex**					
		Female	Ref		Ref	
		Male	1.2 (0.62-2.33)		.58	
**Multivariate logistic regression analysis**						
	**Days after symptom onset**					
		9-10	4.96 (1.2-24)		.03	

## Discussion

### Principal Results

When we compared the performance of the rapid serological test to that of RT-PCR for the detection of SARS-CoV-2 infection, our findings showed that 17.8% (34/191) of the subjects tested positive based on serum IgM/IgG expression, whereas 36.6% (70/191) of the subjects tested positive based on SARS-CoV-2 RT-PCR test results, leading to a sensitivity of 30% and a specificity of 89% for the serological test.

The clinical relevance of so-called rapid serological testing is still an open issue, since the data currently available are still scarce [13]. For this reason, we compared its performance to that of standard RT-PCR testing and analyzed performance with respect to the time of COVID-19–related symptom onset. To this end, we set up a mono-institutional consecutive cohort of patients who were tested with both assays at a single qualified laboratory.

The design of our study allowed us to specifically analyze two aspects of the open issue: (1) the concordance of the rapid serological test results with those of standard molecular testing and (2) the relationship between IgG/IgM expression and the onset of clinical symptoms.

With regard to the degree of concordance between the two tests, the results reported in Figure 1 clearly show that the precision of the VivaDiag rapid test is unsatisfactory. Notably, only 11% (21/191) of the patients that tested positive for COVID-19 based on the molecular test results also tested positive based on the serological test results. This percentage is impressively similar to the performance reported for serological tests in Spain [14] and Germany [15]. However, the first important finding from our study concerns the 6.8% (13/191) of subjects that tested negative based on RT-PCR results, but tested positive based on serological results. The two tests did not produce similar results, which is obvious for assays that are designed to analyze different aspects of COVID-19; the molecular test detects the presence of SARS-CoV-2 in samples based on specific anatomical parts of the respiratory system, while the serology test reveals the kinetics of immunoglobulins as the body reacts to viral infection. Negative serological test results in patients with a positive molecular test could mean that the patients are infected, but have not yet reached the stage of immunoglobulin reaction development. Conversely, subjects who have negative molecular test results, but also have serological test results showing the presence of specific IgG/IgM antibodies, may be recovering from COVID-19. The data shown in Figure 2 seem to confirm these assumptions, as the molecular test yielded more positive results during the early symptomatic phases of the disease in our subjects, while the serological test performed better later on (ie, 10 days after symptom appearance).

The second aspect we were able to analyze in our study was seroconversion and the kinetics of immunoglobulins with respect to the onset of COVID-19–related symptoms. Figure 3 shows the behavior of the two immunoglobulins according to symptom appearance. Interestingly, IgG and IgM did not seem to behave differently based on the number of days elapsing from symptom appearance, but they clearly and progressively increased along the course of the disease. This unexpected finding, in contrast with common knowledge concerning the kinetics of the two immunoglobulins, is supported by the results presented by Zhang et al [16] and Lou et al [17]. Both authors reported that the detectable serology markers, IgG and IgM, had similar seroconversions in COVID-19 patients, with antibody levels increasing rapidly at 6 days after exposure, and this trend occurred with a concomitant decline in viral load. Such behavior in the 6-10-day time window after symptom appearance is typically accompanied by an improvement in serological test sensitivity compared to standard molecular testing.

Very recently, Whitman et al [18] evaluated the performance of 11 SARS-CoV-2 serological assays in a multicentric study that enrolled a cohort of subjects with positive RT-PCR test results. For the serological tests, the incidence of IgG/IgM positivity in the RT-PCR SARS-CoV-2–positive samples ranged from 26.9% to 0%. In particular, the incidence of positive results with the VivaDiag test was 8.2%, which is significantly lower than the value we found in our study. However, the enrollment criteria in the Whitman et al study were very different from ours. In our study, the subjects were consecutively recruited from a single institution, and the tests were performed simultaneously on fresh biospecimens. Interestingly, Whitman et al reported that among the SARS-CoV-2 RT-PCR–positive individuals, the percent seropositivity increased with time, peaking at 81.8%-100.0% in samples taken >20 days after symptom onset. The same trend of increased seropositivity with increased time from symptom onset was observed in our cohort of subjects.

### Limitations

Our study had some important limitations. First, the VivaDiag test was based on the colorimetric evaluation of the IgG and IgM bands performed by the operator, thus implying that all the limitations that a qualitative inter-intra-operator evaluation produces in terms of variability are present in this study [19]. In our study, this was partially solved by resorting to double operator evaluation and taking pictures of all test results to be reanalyzed by a third-party reader in the case of first level evaluation disagreement. Therefore, our next step will be to use quantitative immunoenzymatic methods to analyze SARS-CoV-2–specific immunoglobulins [20] to overcome these issues.

Second, the neutralizing antibodies used in the VivaDiag test might cross-react with other coronavirus antigens, such as those of the SARS-CoV. The recombinant antigen utilized in VivaDiag is the receptor-binding domain of the SARS-CoV-2 spike protein, for which information on possible cross-reactivity with other coronaviruses and flu viruses has not yet been studied [13]. Further studies are urgently needed to definitively clarify this point.

### Conclusions

Our study analyzed the clinical performance of the rapid serological test, VivaDiag, and confirmed the test’s limited applicability for the diagnosis of SARS-CoV-2 infection by comparing its performance to that of standard molecular testing. However, this rapid serological test seems to provide important information concerning individuals’ immunoreaction to the infection, and more importantly, it may detect previous exposure to the virus in currently healthy persons. A trial, recently registered in ClinicalTrial.gov (NCT04316728), will specifically address this issue by investigating the monitoring of seroconversion of COVID-19 IgG/IgM in healthy subjects who may develop COVID-19–related symptoms. In essence, our real-world results should be considered hypothesis-generating findings that warrant further examination in a controlled clinical trial in order to be confirmed [21].

## Abbreviations

IgG: immunoglobulin G
IgM: immunoglobulin M
PCR: polymerase chain reaction
RT-PCR: real-time polymerase chain reaction

## References

[1] Huang C, Wang Y, Li X, Ren L, Zhao J, Hu Y, Zhang L, Fan G, Xu J, Gu X, Cheng Z, Yu T, Xia J, Wei Y, Wu W, Xie X, Yin W, Li H, Liu M, Xiao Y, Gao H, Guo L, Xie J, Wang G, Jiang R, Gao Z, Jin Q, Wang J, Cao B (2020). Clinical features of patients infected with 2019 novel coronavirus in Wuhan, China. Lancet.

[2] Zhu N, Zhang D, Wang W, Li X, Yang B, Song J, Zhao X, Huang B, Shi W, Lu R, Niu P, Zhan F, Ma X, Wang D, Xu W, Wu G, Gao GF, Tan W, China Novel Coronavirus Investigating and Research Team (2020). A Novel Coronavirus from Patients with Pneumonia in China, 2019. N Engl J Med.

[3] Livingston E, Bucher K (2020). Coronavirus Disease 2019 (COVID-19) in Italy. JAMA.

[4] Italy Coronavirus. Worldometer - real time world statistics.

[5] Jin YH, Cai L, Cheng ZS, Cheng H, Deng T, Fan YP, Fang C, Huang D, Huang LQ, Huang Q, Han Y, Hu B, Hu F, Li BH, Li YR, Liang K, Lin LK, Luo LS, Ma J, Ma LL, Peng ZY, Pan YZ, Pan ZY, Ren XQ, Sun HM, Wang Y, Wang YY, Weng H, Wei CJ, Wu DF, Xia J, Xiong Y, Xu HB, Yao XM, Yuan YF, Ye TS, Zhang XC, Zhang YW, Zhang YG, Zhang HM, Zhao Y, Zhao MJ, Zi H, Zeng XT, Wang YY, Wang XH, for the Zhongnan Hospital of Wuhan University Novel Coronavirus Management and Research Team‚ Evidence-Based Medicine Chapter of China International Exchange and Promotive Association for Medical and Health Care (CPAM) (2020). A rapid advice guideline for the diagnosis and treatment of 2019 novel coronavirus (2019-nCoV) infected pneumonia (standard version). Mil Med Res.

[6] Rothe C, Schunk M, Sothmann P, Bretzel G, Froeschl G, Wallrauch C, Zimmer T, Thiel V, Janke C, Guggemos W, Seilmaier M, Drosten C, Vollmar P, Zwirglmaier K, Zange S, Wölfel R, Hoelscher M (2020). Transmission of 2019-nCoV Infection from an Asymptomatic Contact in Germany. N Engl J Med.

[7] Yap JCH, Ang IYH, Tan SHX, Chen JIP, Lewis RF, Yang Q, Yap RKS, Ng BXY, Tan HY (2020). COVID-19 Science Report: Diagnostics. National University of Singapore.

[8] VivaDiag TM SARS-CoV-2 IgM/IgG Rapid Test (COVID-19 IgM/IgG Rapid Test). VivaChek.

[9] Centers for Disease Control and Prevention (2020). Real-time RT-PCR Panel for detection 2019-novel coronavirus : instructions for use. CDC Stacks Public Health Publications.

[10] Landis JR, Koch GG (1977). The measurement of observer agreement for categorical data. Biometrics.

[11] Binnicker M (2020). Emergence of a Novel Coronavirus Disease (COVID-19) and the Importance of Diagnostic Testing: Why Partnership between Clinical Laboratories, Public Health Agencies, and Industry Is Essential to Control the Outbreak. Clin Chem.

[12] Altman DG (1990). Practical Statistics for Medical Research.

[13] Li Z, Yi Y, Luo X, Xiong N, Liu Y, Li S, Sun R, Wang Y, Hu B, Chen W, Zhang Y, Wang J, Huang B, Lin Y, Yang J, Cai W, Wang X, Cheng J, Chen Z, Sun K, Pan W, Zhan Z, Chen L, Ye F (2020). Development and clinical application of a rapid IgM-IgG combined antibody test for SARS-CoV-2 infection diagnosis. J Med Virol.

[14] Elena S (2020). Unreliability of new tests delays effort to slow coronavirus spread in Spain. El Pais.

[15] Schmitt PP (2020). Franffurter Allgemeine. Wir haben neue Symptome entdeckt.

[16] Zhang J, Liu J, Li N, Liu Y, Ye R, Qin X, Zheng R Serological detection of 2019-nCoV respond to the epidemic: A useful complement to nucleic acid testing. medRxiv.

[17] Lou B, Li TD, Zheng SF, Su YY, Li ZY, Liu W, Yu F, Ge SX, Zou QD, Yuan Q, Lin S, Hong CM, Yao XY, Zhang XJ, Wu DH, Zhou GL, Hou WH, Li TT, Zhang YL, Zhang SY, Fan J, Zhang J, Xia NS, Chen Y (2020). Serology characteristics of SARS-CoV-2 infection after exposure and post-symptom onset. Eur Respir J.

[18] Whitman JD, Hiatt J, Mowery CT, Shy BR, Yu R, Yamamoto TN, Rathore U, Goldgof GM, Whitty C, Woo JM, Gallman AE, Miller TE, Levine AG, Nguyen DN, Bapat SP, Balcerek J, Bylsma SA, Lyons AM, Li S, Wong AW, Gillis-Buck EM, Steinhart ZB, Lee Y, Apathy R, Lipke MJ, Smith JA, Zheng T, Boothby IC, Isaza E, Chan J, Acenas DD, Lee J, Macrae TA, Kyaw TS, Wu D, Ng DL, Gu W, York VA, Eskandarian HA, Callaway PC, Warrier L, Moreno ME, Levan J, Torres L, Farrington LA, Loudermilk R, Koshal K, Zorn KC, Garcia-Beltran WF, Yang D, Astudillo MG, Bernstein BE, Gelfand JA, Ryan ET, Charles RC, Iafrate AJ, Lennerz JK, Miller S, Chiu CY, Stramer SL, Wilson MR, Manglik A, Ye CJ, Krogan NJ, Anderson MS, Cyster JG, Ernst JD, Wu AHB, Lynch KL, Bern C, Hsu PD, Marson A Test performance evaluation of SARS-CoV-2 serological assays. medRxiv.

[19] Paradiso A, Ellis IO, Zito FA, Marubini E, Pizzamiglio S, Verderio P (2009). Short- and long-term effects of a training session on pathologists' performance: the INQAT experience for histological grading in breast cancer. J Clin Pathol.

[20] Guo L, Ren L, Yang S, Xiao M, Chang D, Yang F, Dela Cruz CS, Wang Y, Wu C, Xiao Y, Zhang L, Han L, Dang S, Xu Y, Yang QW, Xu SY, Zhu HD, Xu YC, Jin Q, Sharma L, Wang L, Wang J (2020). Profiling Early Humoral Response to Diagnose Novel Coronavirus Disease (COVID-19). Clin Infect Dis.

[21] Camm AJ, Fox KAA (2018). Strengths and weaknesses of 'real-world' studies involving non-vitamin K antagonist oral anticoagulants. Open Heart.

